# Repeated autologous intraarticular blood injections as an animal model for joint pain in haemophilic arthropathy

**DOI:** 10.1186/ar4331

**Published:** 2013-10-07

**Authors:** Michael Karl Boettger, Susanne Krucker, Mieczyslaw Gajda, Hans-Georg Schaible, Thomas Hilberg

**Affiliations:** 1 Institute of Physiology I, University Hospital, Friedrich Schiller University, Jena, Germany; 2 Department of Sports Medicine, University of Wuppertal, Pauluskirchstrasse 7, 42285 Wuppertal, Germany; 3 Current address: Bayer HealthCare AG, Wuppertal, Germany; 4 Institute of Pathology, University Hospital, Jena, Germany

## Abstract

**Introduction:**

Haemophilic arthropathy following recurrent joint bleedings is one of the major disease-related complications in people with haemophilia (PWH), leading to mostly chronic joint pain. Since many antinociceptive principles interfere with the clotting system, PWH are restricted in treatment options, thereby defining a medical need for novel therapeutic principles. However, we lack the availability of an animal model for joint pain in haemophilic arthropathy for testing these.

**Methods:**

In this study, we aimed to validate the rat model of repeated autologous intraarticular blood injections specifically for pain-related behavior. During an observation period of 50 days, groups of animals were injected weekly into one knee joint with either whole blood or cellular/plasma components.

**Results:**

Injections induced primary hyperalgesia starting after the third injection, accompanied by mild functional gait changes and joint swelling. Secondary hyperalgesia and quantitative gait disturbances were not observed. This phenotype was most prominent in whole blood injected animals, with effect sizes of cells and plasma being additive. In order to differentiate haemophilia-related arthropathy from traumatic joint bleeding, another group was injected with whole blood only once, which did not cause any alterations.

**Conclusions:**

Repeated autologous intraarticular injections of blood showed a time course, inflammatory response and reduction in pain thresholds similar to the signs and symptoms observed in PWH. Therefore, this model may be utilised in the future for testing novel antinociceptive principles in haemophilia-associated joint pain.

## Introduction

Haemophilia is the most common genetic clotting disorder and is subdivided into haemophilia A (deficiency in biologically active clotting factor VIII) with a prevalence of 1:10,000, and haemophilia B (deficiency in biologically active clotting factor IX) with a prevalence of 1:50,000 [1]. Since substitution therapy is broadly available, the rate of life-threatening bleedings has dramatically decreased. However, not all bleedings can be avoided, and of these, 85% occur in joints [2], leading to so-called haemophilic arthropathy in the long term. The latter is characterized by cartilage destruction and synovitis which translate into the clinical sign loss of function and symptom joint pain [3,4].

Joint pain in people with haemophilia (PWH) reflects a major clinical problem which starts in early childhood [5]. More than half of the people affected complain about daily joint pain episodes [6], and experimental data have shown a significant reduction of pain thresholds at the joints of PWH [4]. Treatment options for joint pain in PWH are limited, since substances interfering with blood clotting, for example, cyclooxygenase inhibitors, are not unequivocally recommended. Therefore, new therapeutic approaches are warranted. Preclinical testing of such novel antinociceptive principles, however, lacks haemophilia animal models which are specifically validated for the assessment of pain-related behaviour.

We aimed to test whether the previously described model of blood-induced arthropathy in rats [7] may reflect a suitable model for haemophilic joint pain. In particular, this study was designed to answer the following three questions: 1) do animals which are repeatedly injected with blood, display a pain phenotype and can this model thus be validated and used for pain research in haemophilia? 2) what is the relative importance of blood cells and plasma as compared to whole blood on pain-related behaviour and inflammation? and 3) does a single injection of blood, as, for example, observed in joint trauma, cause sustained alterations in inflammation and a pain phenotype, or how does this differentiate from repeated injections?

In order to answer these questions, we simulated joint bleeding by repeated (or single, respectively) homologous intraarticular injections. These were performed either using saline or whole blood, and for the repeated injections isolated plasma or isolated blood cells were also used. In the course of up to 50 days after the first injection, pain-related behaviour and inflammatory changes were assessed in these animals, including measures of primary and secondary hyperalgesia, functional parameters, such as gait and weight bearing analyses, joint swelling and histopathology.

## Methods

### Animals

For the experiments, 63 female Lewis rats (six to eight weeks old, weight 160 to 180 g upon arrival, supplied by Charles River Laboratories, Sulzfeld, Germany) were used. Animals were housed in a 12:12 hour light:dark cycle and received standard rodent chow and water *ad libitum*. Behavioural tests were performed between 8 a.m. and 11 a.m. in a constantly lit and climatized room. All experiments were approved by the Thuringian state authorities (registration number 02-027/10) and complied with EC regulations (86/609/EEC) for the care and use of laboratory animals. Furthermore, the study complies with the ethical guidelines for the assessment of pain in laboratory animals published by the International Association for the Study of Pain [8]. An Extended Methods Form (EMF) for uniform reporting standards as recommended by Rice and coworkers [9] can be found in Additional file 1.

### Treatment protocol and groups

Autologous intraarticular injections were performed repeatedly on days 0, 7, 14, 21, 28, 35, 42 and 49. On each injection day, rats were briefly anesthetized using 2% isoflurane (Isofluran Delta Select, Dreieich, Germany). Blood was drawn from the coccygeal vein after slight warming of the tail using a 100 W infrared lamp (Sanitas SIL5, Dinslage GmbH, Uttenweiler, Germany) using a 26 G cannula (volume: 0.3 ml). The blood or the respective blood component was then immediately injected in the left knee joint of the same animal (autologous blood injection) using a 27 G cannula and an injection volume of 0.1 ml (see below).

In order to differentiate between effects of whole blood (WB), the cellular compartment (white and red blood cells) and the blood plasma, the following groups were investigated: 1) saline injections (n = 9); 2) whole blood injections (n = 9); 3) blood plasma injections (n = 9); 4) injection of cellular blood components (n = 9); and 5) injection of saline containing lithium-heparin (n = 5). For group 2, the blood was directly re-injected. For groups 3 and 4, the obtained blood was transferred into a tube covered with lithium and heparin (Microvette 300 LH, Sarstedt, Nürmbrecht, Germany). The samples were then centrifuged at 2,000 × g at room temperature for five minutes (Heraeus Biofuge 13, Heraeus, Osterode, Germany). For injection of plasma (group 3, cell-free solution), a haematocrit of 40% was assumed, such that 0.04 ml of 0.9% NaCl solution was added to 0.06 ml of plasma. For injection of blood cells without plasma (group 4), the plasma supernatant was removed, and the withdrawn volume was replaced by saline. Of this suspension, again 0.1 ml was injected into one knee joint.

In order to differentiate the effects on pain-related behaviour between (single) traumatic joint bleeding and haemophilic arthropathy, two additional groups were examined in which blood (n = 10) or saline (n = 8) was injected only once (that is, one week after the second baseline testing, groups 6 and 7, respectively) on day 0. In an additional three animals, whole blood was injected weekly according to the aforementioned protocol, and single animals were sacrificed after three, five or eight injections in order to obtain histology (see below) at these time points. One naïve animal was further sacrificed for baseline knee histology (without any injection).

### Pain-related behaviour

Tests for pain-related behaviour were performed twice before the first blood injection in order to acclimatize the animals to the experimental setting and in order to obtain baseline values (for the latter, the values obtained from the second testing were utilized). For all groups, the behavioural tests described below were performed on days 1, 8, 15, 22, 29, 36, 43 and 50 (that is, always one day after blood or blood compartment injection, when performed repeatedly).

#### Stimulus-evoked parameters

Primary mechanical hyperalgesia at the site of the inflamed knee was assessed using a pressure application measurement device (PAM, Ugo Basile, Comerio, Italy) as described previously [10]. In brief, constantly increasing pressure (50 g/second) was applied to the lateral side of the knee joint at the level of the joint space until the animals attempted to escape or vocalized. The weight force to elicit this response was read out in grams. For each animal and testing day, this test was performed once, since repeated testing might further sensitize the nociceptive apparatus.

Secondary mechanical hyperalgesia was assessed in two ways: mechanical thresholds as described above were obtained from the contralateral knee joint using the PAM device. In addition, secondary mechanical hyperalgesia at the paw was assessed using a dynamic plantar aesthesiometer (Ugo Basile) which applied increasing pressure (linear increase in weight force, 2.5 g/second). The weight force eliciting leg withdrawal reflects the respective mechanical threshold. Three consecutive stimuli were applied and the mean force was calculated.

Thermal secondary hyperalgesia at the hind paws was assessed using an algesimeter (Ugo Basile) as described previously [11]. After accommodation of the animals to the testing device, three consecutive standardized heat stimuli were applied to the hind paws with at least two minute intervals between stimuli. Mean latencies were calculated and used as a measure of withdrawal threshold to heat. Stimuli were applied for a maximum of 20 seconds.

#### Functional parameters

Pain-related guarding behaviour of the inflamed hind paw was assessed by quantification of weight bearing towards the non-inflamed hindlimb using an incapacitance tester (Linton Intrumentation, Norfolk, UK). Here, animals were placed in a plastic cage with both hind-paws resting on scales. After accommodation to the device when the animal was sitting calmly, the weight force resting on the two scales was obtained and averaged over three seconds and values from three consecutive measurements were averaged for every testing day. From these values, the relative weight (in %) resting on the inflamed hindlimb was calculated (weight on inflamed hindlimb × 100%/(weight on the inflamed + the non-inflamed hindlimb)) as described previously [12,13].

In addition, a semiquantitative guarding score was assessed as described previously [14]: 0: no guarding, 1: guarding of the hindlimb after a defined brief noxious compression of the knee, 2: visible limping during walking without previous pain stimulus, 3: no use of the hindlimb with the arthritic knee, 4: no movement at all (general morbidity).

For quantitative gait analysis, paw print analyses were used as described previously [15]. Here, animals were gently placed in a piece of cloth and hind paws were stained with liquid dye. Then the rats were placed in the opening of a tunnel, which they entered following their instinct to go to the dark, leaving their paw prints on blotting paper. From these paw prints, the following parameters were obtained: the distance between a print from the left paw and a consecutive print from the right paw or *vice versa* (left-right- or right-left-distance, respectively) and the angle between consecutive paw prints (defined by a line through the incision of the paw print and the third phalanx). For each animal and testing day, at least five artifact-free gait cycles (four consecutive prints) were analyzed and means of these values were used for further analysis.

### Histology and grading of arthritis

Swelling was assessed by measuring the medio-lateral diameter of each knee joint using a vernier caliper (Mitutoyo, Neuss, Germany). For each animal and testing day, the relative swelling was calculated by subtracting the diameter of the non-injected from the injected knee, thus controlling for anatomical knee joint differences between animals.

**Figure 1:**
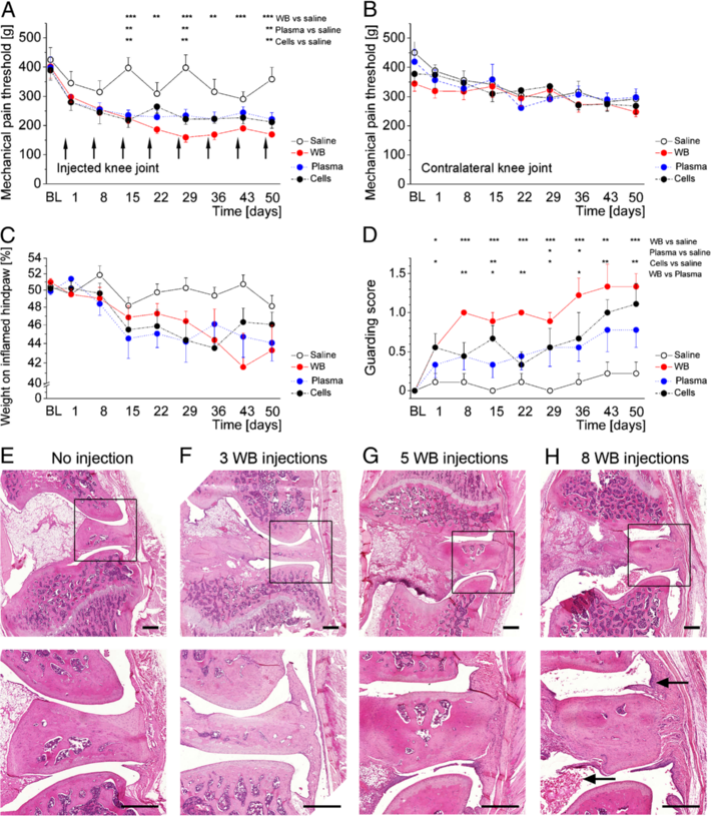
**Time course of pain-related behaviour in repeatedly injected animals.** Thresholds for primary mechanical hyperalgesia assessed at the ipsilateral knee joint were reduced in all blood or blood compartment injected animals compared to those injected with saline **(A)**, arrows indicate the injections for all displayed data), while secondary hyperalgesia obtained from the contralateral knee joint did not show differences between groups **(B)**. The relative weight resting on the injected hindlimb was slightly, yet non-significantly reduced in all groups receiving blood or blood components **(C)**. The guarding score indicating pain- and inflammation-related gait disturbances was significantly increased in all groups, but most prominently in those animals receiving whole blood injections **(D)**. Knee joint histology from different time points is displayed in **(E)** to **(H)**. For comparison, a naïve joint without injections is shown in **(E)**, while joints that received three, five or eight injections, displaying increasing signs of inflammatory changes in the joint space and in surrounding tissues, are shown in **(F)**, **(G)** and **(H)**, respectively. Pictures in the lower panel are magnifications of the indicated inset in the upper panel. Arrows indicate the (mild) inflammatory changes and intra-articular blood cells. Scale bars are 500 μm. Data are presented as means ± SEM. Data of those animals receiving saline in lithium-heparin which were included in the statistical analysis are not displayed in the figure for clarity. * *P* <0.05; ** *P* <0.01; *** *P* <0.001. BL, baseline; SEM, standard error of the mean; WB, whole blood.

**Figure 2:**
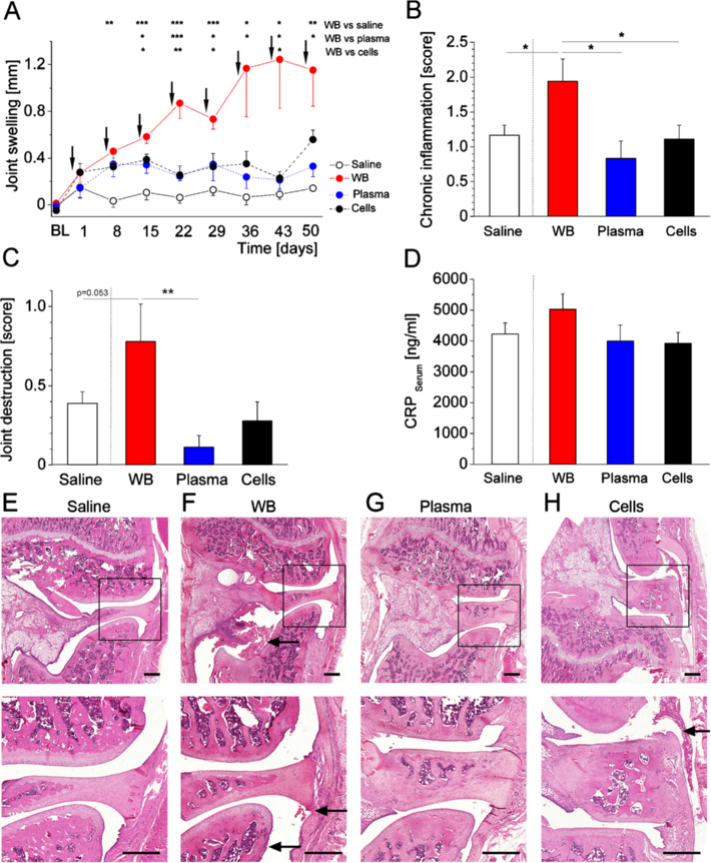
**Inflammatory signs in repeatedly injected animals.** In all parameters assessed, only those animals receiving multiple whole blood injections showed significant signs of inflammation. Joint swelling was significantly increased in this group when compared to all other groups **(A)**. Arrows indicate the injections. Histopathological signs of chronic inflammation were likewise significantly increased in whole blood injected animals, but not those receiving blood compartments **(B)**, while signs for joint destruction only showed a trend **(C)**. Numerically, C-reactive protein levels were highest in whole blood injected animals, yet here, no statistical difference could be obtained between groups **(D)**. Representative pictures of knee joint histology from the different groups are displayed in **(E)** to **(H)**. An animal that received eight saline injections is shown in **(E)**. Here, no inflammatory response is obvious. A specimen from a whole blood injected animal is displayed in **(F)**, showing signs of synovitis and inflammation in the surrounding tissues as well as blood cells in the joint space. Plasma-injected animals **(G)** and those that received cellular compartments are shown in **(H)**, revealing similar, yet milder pathology compared to whole blood. Pictures in the lower panel are magnifications of the indicated inset in the upper panel. Arrows indicate the (mild) inflammatory changes and intra-articular blood cells. Scale bars are 500 μm. Data are presented as means ± SEM. Data of those animals receiving saline in lithium-heparin which were included in the statistical analysis are not displayed in the figure for clarity.* *P* <0.05; ** *P* <0.01; *** *P* <0.001. BL, baseline; SEM, standard error of the mean; WB, whole blood.

Histology of the knee joints was assessed on day 50 (except for those animals that were sacrificed after three or five injections, see above). Rats were deeply anesthetized with 120 mg/kg sodium thiopentone i.p. (Trapanal, Byk Gulden, Konstanz, Germany) and sacrificed. The knee joints were removed, skinned, post-fixed in formalin, decalcified in 7% AlCl_3_ (in 2.1% HCl and 6% formic acid) for 48 hours**,** embedded in paraffin, cut into 5 μm thick frontal sections, and stained with haematoxylin-eosin. Two independent observers unaware of the treatment scored the sections (0: no, 1: mild, 2: moderate, 3: severe alterations). The amount of fibrin exudation, the relative number and density of granulocytes in synovial membrane and joint space allowed grading of the acute inflammatory reaction, and the relative number and density of infiltrating mononuclear leukocytes in the synovial membrane, the degree of synovial hyperplasia, the extent of infiltration and fibrosis in the peri-articular structures allowed grading of chronic inflammation. Cartilage and bone destruction (taken together as ‘joint destruction’) were also scored (0: no erosion, 1: erosion of <10%, 2: of 10% to 25%, 3: of 25% to 50%, and 4: of >50% of cartilage and bone in cross sections) [14,16]. Histological pictures for Figures 1 and 2 were obtained using Mirax Scan 150 (Zeiss, Jena, Germany).

### Statistical analyses

For statistical analyses, SPSS for Windows (version 17.0) was used. First, all data were tested for normal distribution applying Kolmogoroff-Smirnoff-tests.

In order to test significant group differences over time in those animals receiving repeated injections, repeated measures analysis of variance (ANOVAs) were performed with the between subjects factor ‘GROUP’ consisting of groups 1 to 5, and the within subject factor ‘TIME’ (baseline, days 1, 8, 15, 22, 29, 36, 43 and 50). *Post-hoc* t-tests were only performed when ANOVAs showed a significant GROUP X TIME interaction. Animals injected with saline briefly incubated in lithium heparin were included in the statistical analyses, yet their data are not displayed in the figures, since these were mostly identical to those who were injected with saline.

In order to test for significant group differences between single blood injection and single saline injection, repeated measures ANOVAs for all parameters were performed with the between subjects factor ‘GROUP’ (blood versus saline, that is, group 6 versus group 7) and the within subject factor ‘TIME’ (baseline, days 1, 8, 15, 22, 29, 36, 43 and 50). Again, whenever ANOVAs revealed a significant GROUP X TIME interaction, *post-hoc* t-tests were calculated for descriptive analysis.

Group differences in histopathological scores obtained from the tissues removed on day 50 were statistically evaluated using *Χ*^2^ tests, since, here, no normal distribution could be established due to the categoric nature of the data. Follow-up Mann–Whitney-tests were then used for descriptive analysis. Statistical significance was assumed for *P* <0.05.

## Results

### Effects of repeated whole blood and blood compartment injections on pain-related behaviour

When comparing pain-related behaviour during the observation period of 50 days, a significant main effect (GROUP X TIME interaction) could be observed for primary hyperalgesia as obtained from applying increasing pressure to the blood-injected knee joint (F(32,105) = 1.736; *P* = 0.020). Repeated saline injections led to a slight, but non-significant decrease in pressure pain thresholds, while repeated whole blood injections showed significantly decreased thresholds from day 15 onwards. Both plasma and cellular compartment injections showed a similar decrease in thresholds up to day 15, but remained on a slightly higher level until day 50 (see Figure 1A). Secondary hyperalgesia as measured by mechanical pain thresholds at the contralateral knee (PAM device) was not seen in any of the groups (F(32,105) = 0.684; *P* = 0.890, Figure 1B). Likewise, no secondary mechanical or thermal hyperalgesia could be detected upon filament stimulation (dynamic plantar aesthesiometer) or radiating heat (Hargreave’s method), respectively, applied to the paws (secondary mechanical hyperalgesia injected side F(32,105) = 1.095; *P* = 0.356 and contralateral side F(32,105) = 1.028; *P* = 0.442; secondary thermal hyperalgesia injected side F(32,105) = 0.699; *P* = 0.876 and contralateral side F(32,105) = 1.045; *P* = 0.419, data not shown).

Assessment of weight bearing showed a decrease from 50% body weight resting on the injected hind paw at baseline to approximately 42% to 45% from day 15 onwards in all blood or blood compartment injected groups (see Figure 1C), yet statistical analysis did not reveal a significant GROUP X TIME interaction (F(32,105) = 1.084; *P* = 0.370).

Gait analysis revealed significant differences between groups for the parameter guarding score (F(32,105) = 2.159; *P* = 0.002). Here, most obvious disturbances of gait were observed in those animals that received whole blood injections and, to a lesser degree, in those animals receiving either plasma or cellular components (Figure 1D). Interestingly, these changes were the first to be observed, starting on day 1 after the first injection, reaching a plateau from day 36 onwards for whole blood. Quantitative gait analysis, however, did not reveal significant differences between groups. Here, outward rotation (angle between paws) was unaltered, while stride length (distance from left to right paw print) tended to decrease towards later observation time points, but did not reach statistical significance (F(32,101) = 1.256; *P* = 0.113 and F(32,101) = 1.183; *P* = 0.261, respectively, Table 1, data for contralateral side not shown).

**Table 1:** Quantitative gait parameters (injected side)

**Distance left to right paw (stride length) [cm]**									
Test day	BL	1	8	15	22	29	36	43	50
Saline	5.4 ± 0.1	5.2 ± 0.3	4.9 ± 0.4	5.2 ± 0.2	4.9 ± 0.4	4.9 ± 0.3	5.2 ± 0.3	4.9 ± 0.3	5.5 ± 0.2
WB	5.8 ± 0.2	5.8 ± 0.4	5.3 ± 0.3	5.0 ± 0.3	4.9 ± 0.2	5.0 ± 0.4	4.5 ± 0.3	5.1 ± 0.5	4.9 ± 0.3
Plasma	5.6 ± 0.2	5.1 ± 0.3	5.3 ± 0.3	5.2 ± 0.3	5.1 ± 0.3	4.3 ± 0.3	4.5 ± 0.2	4.5 ± 0.3	4.4 ± 0.4
Cells	5.9 ± 0.2	5.4 ± 0.3	5.4 ± 0.3	5.4 ± 0.1	4.9 ± 0.3	4.8 ± 0.2	4.5 ± 0.4	5.0 ± 0.3	4.1 ± 0.3
**Angle between paws (outward rotation) (°)**									
Test day	BL	1	8	15	22	29	36	43	50
Saline	30 ± 2	31 ± 2	31 ± 4	31 ± 3	28 ± 2	29 ± 2	31 ± 2	31 ± 2	30 ± 3
WB	32 ± 2	33 ± 3	32 ± 2	34 ± 2	32 ± 2	32 ± 2	36 ± 2	34 ± 1	32 ± 1
Plasma	32 ± 2	31 ± 2	32 ± 2	31 ± 1	32 ± 2	35 ± 2	34 ± 1	35 ± 3	33 ± 2
Cells	34 ± 2	37 ± 2	33 ± 2	35 ± 2	35 ± 2	32 ± 2	33 ± 2	36 ± 2	36 ± 2

Data are presented as mean ± SEM. There were no significant differences between groups. BL, baseline; SEM, standard error of the mean; WB, whole blood.

A similar time course as for the development of pain-related behaviour could be observed for the histological changes in the affected knee joints (Figure 1E-H). Here, in naïve animals no alterations of the joint and no signs of inflammation can be observed (Figure 1E). After three whole blood injections (corresponding to day 15), there is a slight inflammatory reaction of the lining cells, but no gross pathology (Figure 1F). From the fifth whole blood injection onwards (corresponding to days 29 and above), blood cells remain visible in the joint space, and an inflammatory reaction of the synovia, but also of surrounding tissues can be observed (Figure 1G,H).

### Effects of repeated whole blood and blood compartment injections on inflammation and joint destruction

Joint swelling showed significant differences between groups over time (F(32,105) = 2.304; *P* <0.001). Here, both plasma and cellular compartments showed slightly, yet non-significantly, increased joint diameters as compared to saline injections, while whole blood injections led to significantly increased joint swelling from day 8 onwards, with approximately four-fold total levels as compared to blood compartments at the end of the observation period (Figure 2A). Histological analysis of the joints obtained on day 50 mirrored these findings, since here, scores for chronic inflammatory changes were significantly increased in whole blood injected animals compared to saline, but not in those animals receiving blood compartments (*Χ*^2^ = 12.558; *P* = 0.014, Figure 2B). In tendency, the same pattern was observed for joint destruction (*Χ*^2^ = 13.456; *P* = 0.009, Figure 2C). Histological sections of representative animals from the different groups (saline, whole blood, plasma and cells) are displayed in Figure 2E-H.

The inflammatory marker C-reactive protein obtained from venous blood on day 50 was slightly increased in whole blood injected animals, yet one way ANOVA did not reveal significant differences between groups (F = 1.321; *P* = 0.285, Figure 2D).

### Effects of a single whole blood injection on pain-related behaviour, inflammation and joint destruction

**Figure 3:**
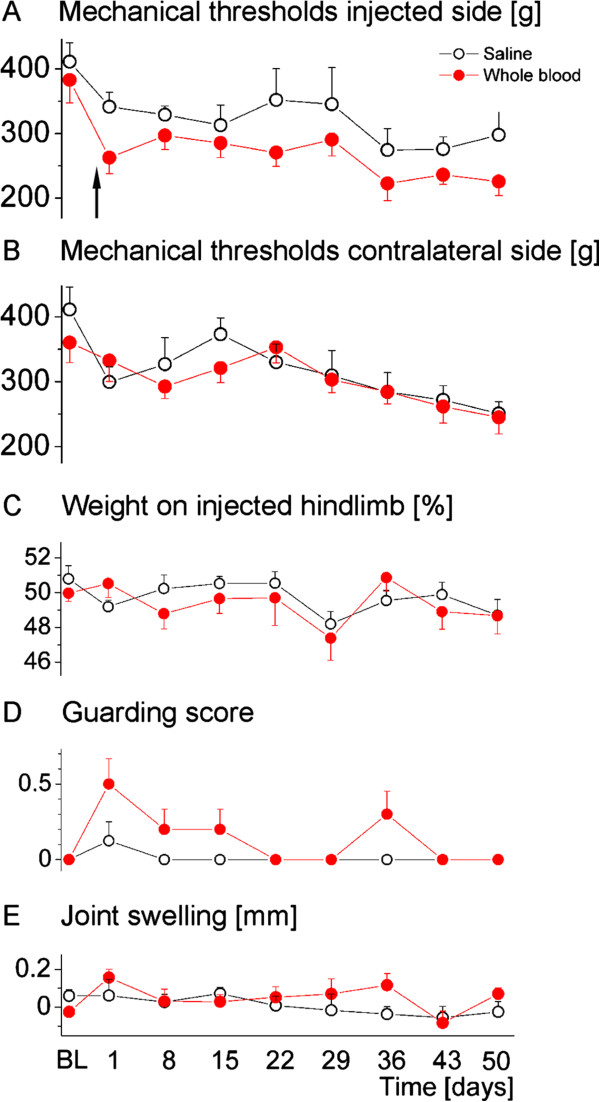
**Parameters of pain-related behaviour and inflammation in animals injected with saline or whole blood once on day 0 (arrow).** No differences were obvious for primary hyperalgesia **(A)**, secondary hyperalgesia **(B)**, weight bearing **(C)**, guarding score **(D)** or joint swelling **(E)**. Data are presented as means ± SEM. BL, baseline; SEM, standard error of the mean.

A single intraarticular injection of whole blood was compared to a single saline injection. Measures of primary (F(8,9) = 0.52; *P* = 0.818) and secondary hyperalgesia (F(8,9) = 2.42; *P* = 0.105) were not different between groups (Figure 3A,B). Likewise, there was no difference in weight bearing (F(8,9 = 1.19; *P* = 0.397) (Figure 3C). Despite a slight increase in the guarding score on day 1 which was not significantly different from saline, there were no signs of gait disturbances (F(8,9) = 2.39; *P* = 0.113) (Figure 3D, quantitative analyses not shown). Likewise, there was no difference between groups regarding joint swelling (F(8,9) = 3.07; *P* = 0.057) (Figure 3E) or histologically assessed inflammation and joint destruction (not shown). Thus, in summary, a single whole blood injection did not cause persisting changes over time.

## Discussion

At present, no animal models of blood-induced arthropathy have been specifically validated regarding haemophilic or blood-induced joint pain. We employed a battery of tests to characterize pain-related behaviour in a rat model of autologous intraarticular injections as originally described by Niibayashi and coworkers [7].

While a single intraarticular injection of whole blood led to very mild, non-significant and reversible pain-related behaviour, repeated injections resulted in significant and sustained primary hyperalgesia and an increase in guarding, as well as a trend for weight bearing alterations. This pain-related behaviour became obvious following the third injection. Secondary hyperalgesia and quantitative gait parameters were not altered, even after multiple injections. Injections of blood compartments led to a similar, yet clearly milder pain phenotype. In whole blood injected animals, a significant inflammatory response could be observed also. Overall, this model may serve to test novel treatment principles for haemophilic arthropathy and related pain states. However, this may be limited to different routes of administration and compound classes. Since there is no genetic factor VIII deficiency, supplementation regimes cannot be evaluated in this model. Furthermore, topically injected compounds should be considered with caution, since these may interfere with the model itself. However, for any route of administration other than intra-articular injection, the model appears robust and viable.

### Choice of the model

In haemophilia research, several animal models have been described. These include genetic models in dogs (for example, Chapel-Hill colony for haemophilia A, [17]) and mice [18] or the injection of inhibitors or of antibodies against clotting factors or their receptors [19-21]. In all of these models, however, hardly any spontaneous bleedings into joints are seen [22] and, therefore, they need to be induced, for example, by blunt trauma [23]. For the examination of localized phenomena induced by bleedings, injections of blood in dogs and rats have previously been utilised [7,24]. For our purpose, we chose the latter since, here, bleedings can be simulated in a standardized fashion and variability between animals can thus be kept small (time course, regular testing, blood volume). Furthermore, joint alterations similar to haemophilic arthropathy have been described [7] in this model. Finally, this study design allowed us to examine the isolated effects of different blood components. Certainly, this choice limits our study with respect to factor VIII/IX content and related clotting disorders which are not likewise simulated. Since in the genetic models, however, traumatic impact is needed, results from such a setting are likewise biased. Despite more data being available for the respective canine model, we used rats since, for this species, the employed behavioural tests are best validated.

Employing this model, we could establish primary hyperalgesia starting from day 15, following the third whole blood, plasma or cell injection. At the same time, the first mild signs of synovitis become obvious which aggravate with additional injections. From other studies it is known that other signs of haemophilic arthropathy also become evident only after repeated injections/bleedings [25,26]. Moreover, while acute injury with haemarthrosis in mice deficient for factor VIII immediately leads to a transient loss of locomotor performance as assessed using a rotarod, those animals that, in fact, develop synovitis after three injuries show a delayed, but highly significant alteration of their locomotor behaviour which may be influenced by both pain and joint destruction [27].

Interestingly, we found a reduction of pressure pain thresholds by 50% which mirrors those seen in people with haemophilia [4], thereby matching the clinical situation. In contrast to more aggressive models of rheumatoid arthritis such as collagen-induced arthritis or antigen-induced arthritis [15,28], however, we were not able to detect any mechanical or thermal secondary hyperalgesia, that is, reduced pain thresholds in areas remote from the injected joint. The observed joint pain and inflammation further led to slight functional alterations, best depicted in the guarding score which quantifies limping of the animals. Quantitative gait parameters were not significantly affected, despite a trend towards a reduction in stride length in the late observation phase. This again translates well to the clinical situation, where a discrete reduction of stride length was also observed using elaborate methodology [29]. The inflammatory changes observed in this study are comparable to those described in the literature for repeated intraarticular blood injections in animal models [22,30] as well as to the synovitis and joint destruction seen in PWH [31].

### Relative contribution of blood compartments

Several factors have been identified which contribute to haemophilic arthropathy or blood-induced joint disease [2,32]. To date, however, it is not entirely clear which blood components drive blood-induced joint disease in different stages. There is convincing evidence that increased iron concentrations following erythrocyte degradation play a significant role. Here, heme, hemosiderin and/or heme-derived iron are considered toxic, since they can mediate oxidative stress and inflammation [32-34]. In addition, white blood cells, particularly mononuclear cells, have been implicated in haemophilic arthropathy. They can enter the joint either by the bleeding itself or secondarily due to chemotaxis, following an initial inflammatory response of the cartilage tissue [32]. Upon activation by synovial triggering, these cells then release, besides other cytokines and inflammatory mediators, IL-1β and TNF-α which can then maintain and exacerbate the inflammatory response, similar to other arthritic conditions [35]. Finally, blood plasma components have also been shown to add to the pathophysiology. For instance, thrombin and fibrin have been shown to potentially exacerbate inflammatory responses in haemophilic joint conditions [36,37]. A different explanation for long-term arthropathy in haemophilia, following multiple bleedings, may be a secondary development of auto-immune joint disease. Recently, Murakami and coworkers could show that repeated microbleedings could facilitate autoimmune arthritis in an animal model, thereby aggravating inflammation [38].

Despite this knowledge of the overall pathophysiology of blood-induced joint damage, little is known about the relative contribution of these components to the generation of joint pain. Of the mechanisms described, most data exist for the role of pro-inflammatory cytokines in joint pain [39]. In this study, we thus aimed at differentiating those effects caused by whole blood or isolated plasma or cellular components. Here, the injection of whole blood caused the most severe pain phenotype, and also the strongest inflammation, thereby suggesting a role for cytokine-induced peripheral sensitization of nociceptors. Furthermore, our data show an additive effect of both cells and plasma on pain-related behaviour and inflammation, with whole blood injections revealing the strongest effects. This is somewhat in contrast to *in vitro* studies investigating inhibition of proteoglycan synthesis which showed nearly maximal effects when erythrocytes and mononuclear cells were incubated with chondrocytes, whereas addition of plasma did not cause further aggravation [40,41]. It is not clear, however, to what extent proteoglycan synthesis and nociception in the knee joint may be linked.

### Single whole blood injection

In order to differentiate multiple intraarticular bleedings reflecting a model for haemophilic arthropathy from traumatic joint bleeding, as clinically mostly observed in anterior cruciate tears [42], we also examined one group receiving a single injection. Here, despite a small, non-significant increase in guarding early after the injection, no gross abnormalities could be observed. This is well in line with data from a canine study, in which dogs showed nearly normal weight bearing already one hour after intraarticular joint injection [24]. The discrepancy between a usually painful haemarthrosis in patients and the absence of pain-related behaviour in this model can be explained by the blood volume in the knee joint. After trauma, blood usually fills the entire joint cavity, while here, the volume was restricted. The main question to be answered in this study was whether a single (comparable) injection of whole blood would induce long-term changes, as might be expected from the pathomechanisms described above [32] and from *in vitro* studies [40,43]. This is clearly not the case and is, therefore, in line with previous *in vivo* studies which suggested long-term bleeding-related joint damage only occurs when the trauma also destabilizes the knee joint, for example, by ligament rupture [24,44].

### Limitations

In addition to considerations regarding the choice of the model (see above), one additional potential limitation needs to be addressed: for the examination of blood compartments, we chose always to inject the same volume and aimed at maintaining the same relative content of cells/plasma as in whole blood. This means that the cells were re-suspended in saline and that the plasma was diluted in saline prior to injection. This may have artificially reduced the efficacy of the two compartments and, thus, may explain differing findings from a canine study, in which the cellular components alone were shown to exhibit the same effects as whole blood [45]. In this particular study, however, cells were concentrated up to the injection volume. In contrast, our approach was to inject the same total content of cells/plasma into the joint.

## Conclusions

In this study, we validated the rat model of repeated autologous intraarticular injections of blood as a model for joint pain in PWH. In particular, the time course, the need for repeated injections/bleedings, the concomitant inflammatory response and, finally, the relative reduction in pressure pain thresholds are comparable to those signs and symptoms observed in PWH. Mechanistically, blood cells and plasma need to be present in the joint to induce the full picture of haemophilic arthropathy, displaying mostly additive effects. Therefore, this model may be utilised in the future for testing novel antinociceptive principles in haemophilia-associated joint pain.

## Abbreviations

ANOVA: Analysis of variance; BL: Baseline; EMF: Extended method form; IL-1β: Interleukin-1 beta; PAM: Pressure application measurement; PWH: People with haemophilia; SEM: Standard error of the mean; SPSS: Statistical package for the social sciences; TNF-α: Tumor necrosis factor alpha; WB: Whole blood.

## Competing interests

The authors declare that they have no competing interests.

## Authors’ contributions

MB designed the study, organized funding, supervised the experiments, analyzed the data and wrote the manuscript. SK performed the *in vivo* experiments, analyzed the data and incorporated these in the manuscript. MG performed the histological analysis of tissues, analyzed the data and incorporated these in the manuscript. HGS supervised the study and wrote the manuscript. TH designed the study and wrote the manuscript. All authors read and approved the final manuscript.

## Supplementary Material

Additional file 1 Extended Methods Form. Click here for file
