# Supplementary material for: Repeated autologous intraarticular blood injections as an animal model for joint pain in haemophilic arthropathy
Source: Arthritis Res Ther. 2013 Oct 7;15(5):R148. doi: 10.1186/ar4331 (PMC3978931; doi:10.1186/ar4331)
Supplement: Additional file 1 — Extended Methods Form. [file ar4331-S1.pdf]

Extended **M**ethods **F**orm

Rice ASC, Cimino-Brown D, Eisenach JC, Kontinen VK, LaCroix-Fralish ML, Machin I, Mogil JS, Stöhr T. Animal models and the prediction of efficacy in clinical trials of analgesic drugs: a critical appraisal and call for uniform reporting standards. *Pain 2008;139(2):243-7*

1. Experimental design

Experiments blinded	Yes
- what	Pain Condition
- how	Experimenter unaware of compound injected
Randomization	Yes
Power calculation	Yes
How many data points removed?	1-5
- why	Animals revealed abnormal

2. Animals

Age	Young Adult
Sex	Female
Genetic background of mutation	Lewis
Supplier	Charles River, Germany
Weight Range	160 - 180 g upon inclusion
Behavioral Abnormalities	none

Any additional comments	

3. Environment

a. *Housing*

Diet	Standard rodent chow and
Bedding	Standard rodent bedding
Cage rack ventilation	Not ventilated (std. cage)
Home cage enrichment	None
Habituation time before experiments (in days)	>7 days
Handling frequency before testing	Once
Cage cleaning frequency	Twice per week
# housed per cage	4
House experimental/ control groups together or separately	Different cages
Different species housed in same room	No
Lights on time	7:00
Lights off time	19:00
Procedural anesthetic	Inhal. Isoflurane
Post-operative analgesia	No
-Analgesic and dose	

b. *Testing*

# of testers	1
Experience of tester(s)	6 months - 1 year
Lighting intensity (lux)	not determined
Time testing ends	11:00
Test environment noise	Quiet
Recent calibration of testing equipment	Yes
Cleaning of test equip. between subjects	Yes
-with	Water
Habituation time before testing (in minutes)	10-30 minutes
Oestrus stage of females	Unknown
Number of animals present in testing room	10
Distance separated	2-3 m
In visual contact	Yes
Arousal state	Not sleeping

Comments on this form to: Andrew Rice *a.rice@imperial.ac.uk*
